# Pharmacodynamics of rituximab on B lymphocytes in paediatric patients with autoimmune diseases

**DOI:** 10.1111/bcp.13970

**Published:** 2019-06-20

**Authors:** Shan Pan, Huixin Yu, Ayesha Surti, Iek Cheng, Stephen D. Marks, Paul A. Brogan, Despina Eleftheriou, Joseph F. Standing

**Affiliations:** ^1^ UCL Great Ormond Street Institute of Child Health London UK; ^2^ Novartis Pharma AG Basel Switzerland; ^3^ UCL School of Pharmacy London UK; ^4^ Great Ormond Street Hospital for Children NHS Foundation Trust London UK; ^5^ Guy's and St Thomas' NHS Trust London UK

**Keywords:** autoimmune diseases, B lymphocytes, NONMEM, paediatrics, pharmacodynamics, rituximab

## Abstract

**Aims:**

Rituximab is a chimeric IgG‐1 monoclonal antibody that depletes B cells, aiding in the treatment of several conditions including autoimmune diseases. It is not licensed for use in children. This study aimed to quantify the B cell‐related pharmacodynamics of rituximab in children with autoimmune disease.

**Methods:**

Routine electronic health record data were collected at a large paediatric tertiary hospital in London, UK. Dosing protocols were either 2 × 750 mg/m^2^ intravenous infusions of rituximab on days 1 and 15, or 4 × 375 mg/m^2^ infusions on days 1, 8, 15 and 22. Rituximab pharmacokinetics (PK) were not measured but CD19+ lymphocyte counts were taken before and after rituximab treatment. A dose–response model was constructed describing the life cycle of CD19+ lymphocytes, with rituximab assumed to increase the death rate. Rituximab effect was assumed to decay by first‐order kinetics.

**Results:**

In total, 258 measurements of CD19+ lymphocyte counts were collected from 39 children with 8 autoimmune diseases. The elimination rate constant (% relative standard error) of rituximab effect decay was 0.036 (22.7%) days^−1^ and CD19+ turnover was 0.02 (41%) days^−1^ corresponding to half‐lives of 19 and 35 days respectively. Rituximab increased CD19+ death rate 35‐fold, with methotrexate and cyclophosphamide associated with further increases. Simulations suggested that a single infusion of 750 mg/m^2^ provides similar 6‐month suppression of CD19+ lymphocytes to current dosing.

**Conclusions:**

Rituximab pharmacodynamics (PD) in paediatric autoimmune diseases has been described. Compared with rituximab alone, the additional effect of methotrexate or cyclophosphamide was statistically significant but small.

**What is already known about this subject**

- Rituximab is unlicensed for paediatric autoimmune diseases, but frequently used off‐label.
- Rituximab pharmacokinetics have been described in children in oncology, but few pharmacokinetic or pharmacodynamic data are available in children with autoimmune diseases.

**What this study adds**

- The turnover half‐life of CD19+ lymphocytes and drug effect decay were similar in paediatric patients with autoimmune diseases than those observed in other populations.
- Current dosing schemes could be reduced by half and still give a similar degree of CD19+ suppression

## 1 INTRODUCTION

Rituximab is a chimeric murine/human monoclonal antibody that was first approved in 1997 by the US Food and Drug Administration for the treatment of non‐Hodgkin's lymphoma. Later, approval of rituximab was granted for the treatment of post‐transplant lymphoproliferative disorder and several autoimmune diseases including rheumatoid arthritis, granulomatosis with polyangiitis and other anti‐neutrophil cytoplasmic antibody‐associated vasculitides.^1^ Rituximab is not licensed for use in paediatric patients but has been administered off‐label for autoimmune diseases such as juvenile systemic lupus erythematosus and lupus nephritis, anti‐neutrophil cytoplasmic antibody‐associated vasculitis, and rheumatoid factor positive juvenile idiopathic arthritis as well as nephrotic syndrome before and after transplantation and for induction and prophylaxis in antibody incompatible transplantation. Rituximab doses are individually adjusted by body surface area, although the impact of different doses and disease control in paediatric autoimmune disease is unknown.

Rituximab functions through depleting B cells that are considered to be major players in regulating immune responses to pathogens and autoantigens,^2^ and hence has been used for controlling a range of B‐cell malignancies including autoimmune diseases. The mechanism of action of rituximab is specific binding to the cluster of differentiation (CD) 20 antigen on the surface of B cells, which is a differentiation marker exclusively expressed in mature B cells.^3^ In addition, rituximab‐mediated B lymphocyte clearance may act via a number of mechanisms, including complement‐mediated toxicity, antibody‐dependent cell‐mediated cytotoxicity, inhibition of cell proliferation and induction of apoptosis.^3^ In assessing B cell counts, it is usual that CD19 surface antigens are measured, as CD19 is expressed continuously from the early pre‐mature B cells to the early stages of plasma cell differentiation.^2^

A single course of rituximab administration consists of 2 sets of intravenous infusions at 750 mg/m^2^ given twice 2 weeks apart or 4 weekly infusions of 375 mg/m^2^ given weekly. Treatment courses may be repeated based on clinical response. After administration, like most monoclonal antibodies, rituximab distributes and binds to FcRn receptors on the surface of endothelial cells to be protected from lysosomal degradation, which results in a long elimination half‐life.^4^ Following rituximab treatment, the B‐lymphocyte depletion effect occurs rapidly, within 2 weeks, and persists for up to 6 months.^5^, ^6^

Whilst rituximab appears on the 2015 World Health Organisation Essential Medicines List, it costs £3493 per course in the UK^7^ and adds to the financial burden facing the National Health Service (NHS). Biosimilars of rituximab are now available with the cost savings of at least 10%.^8^ Bioequivalence of biosimilars with respect to reference biologics needs to be judged by pharmacokinetic–pharmacodynamic (PKPD) principles requiring established PKPD knowledge, rather than the case with small molecules whereby PK‐only equivalence is required. To date, there are few PK studies^9^ and to our knowledge no PD studies reporting the use of rituximab in paediatric patients with autoimmune diseases.

This study therefore aimed to develop a mathematical model of the effect of rituximab on eliminating B lymphocytes when used to treat autoimmune diseases in children. The model was then used to investigate covariates associated with response and dosing regimens of rituximab that provide adequate suppression of B lymphocytes whilst minimising the dose.

## 2 METHODS

### 2.1 Data source

In the period from 1 January 2010 to 31 December 2016, all children treated with rituximab for autoimmune diseases were identified from the pharmacy prescribing database at Great Ormond Street Hospital for Children NHS Foundation Trust, London, UK.

For these patients, data on concomitant medications, basic demographics (diagnoses, age, sex) and CD19+ lymphocyte counts in peripheral blood measured by flow cytometry were collected. Ethical approval without the need for parent and patient consent was obtained for the retrospective analysis of the de‐identified data (17/LO/0008).

### 2.2 Population kinetic–PD modelling

**Figure 1:**
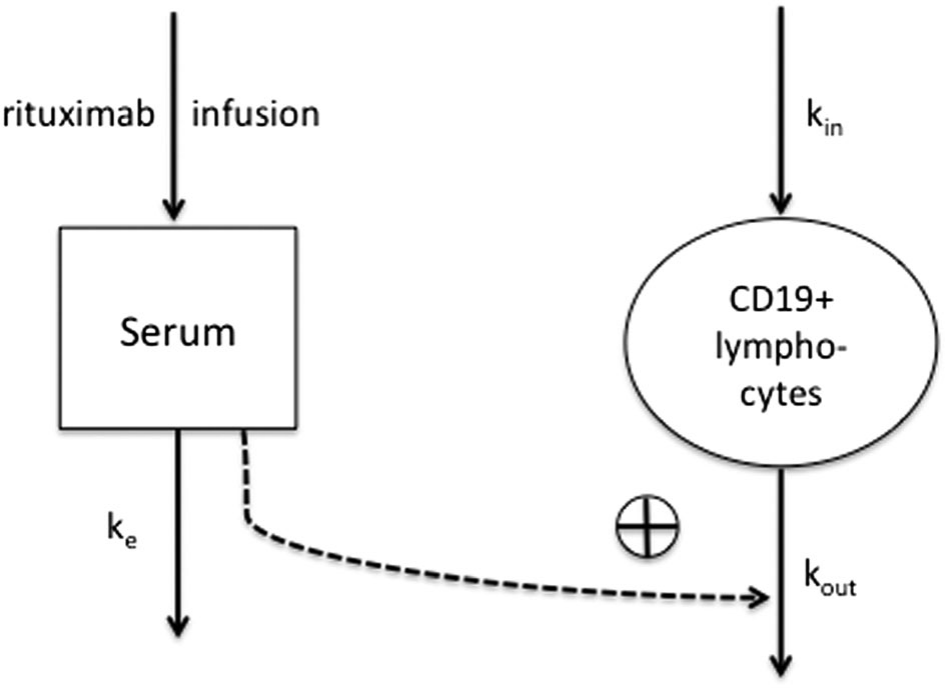
Diagram of rituximab kinetic–pharmacodynamic model. k_e_, elimination rate constant of rituximab; k_in_, production rate of CD19+ lymphocytes; k_out_, elimination rate constant of CD19+ lymphocytes; plus sign, promotion on CD19+ lymphocytes elimination via E(t) = (E_max_*D(t))/(ED_50_ + D(t)); E(t), promotion of cell killing effect at time t; E_max_, maximum promotion effect; ED_50_, dose producing 50% of the maximum promotion effect; D(t), rituximab residual dose within the body at time t)

A dose–response model for describing the change of CD19+ lymphocyte counts after rituximab treatment was constructed in NONMEM (version 7.3.0). First‐order decay in apparent rituximab effect was assumed. A turnover model was constructed to describe the life cycle of CD19+ lymphocytes considering the effect of rituximab on increasing the death rate of CD19+ lymphocytes. A schematic diagram of the kinetic–pharmacodynamic (K‐PD) model is shown in Figure 1. Equations for the K‐PD model are written as below:

(1) $${\mathrm{dA}}_{1}/\mathrm{dt}=-k_{e}\times A_{1}$$

(2) $${\mathrm{dA}}_{2}/\mathrm{dt}=k_{\text{in}}-k_{\text{out}}\times \left(1+E_{\max}\times A_{1}/\left({\mathrm{ED}}_{50}+A_{1}\right)\right)\times A_{2}$$

Here, A_1_ has initial conditions of 0, with addition of a dose amount in mg added with every dose event, and k_e_ representing elimination rate constant of rituximab effect, k_in_: production rate of CD19+ lymphocytes, k_out_: elimination rate constant of CD19+ lymphocytes, E_max_: maximum increase in cell elimination rate in the presence of rituximab, ED_50_: dose at which produces 50% of the maximum effect, A_2_ represents CD19+ lymphocytes. In the absence of rituximab at steady‐state the baseline CD19+ count is given by k_in_/k_out_ and we estimated baseline and k_out_ as parameters, from which k_in_ was derived. The baseline parameter was used to initialize the CD19+ lymphocytes compartment. NONMEM code is provided in the supplementary material. Proportional, additive and combined additive and proportional residual error models were tested.

The following covariates were tested on the K‐PD model parameters: age, sex and comedications (methotrexate, cyclophosphamide, azathioprine or mycophenolate mofetil). Other concomitant immunosuppressants were not considered since they were either taken by all patients (e.g. prednisolone or methylprednisolone) or by very few patients (e.g. 2 patients on tacrolimus) and their effects on CD19+ lymphocytes were unable to be identified. Since B‐cell count is expected to fall with age,^10^ asymptotic decrease in CD19+ lymphocyte production rate over age was examined via adding age on the k_in_ parameter. Whether female children possessed higher CD19+ lymphocyte production rate was also tested.^10^ Individual comedications were considered to increase apparent E_max_ or to decrease apparent ED_50_.

A covariate was considered statistically significant when the reduction in –2log likelihood of the model was >3.84 for an additional 1 degree of freedom at the significance level of 0.05 using the likelihood ratio test.^11^

CD19+ lymphocyte counts reported as zero were set to 5 × 10^6^/L (half the lowest detectable). Both M3 and M6 (substituting zero counts with the lower limit of quantification [LLOQ] divided by 2 [LLOQ/2]) methods^12^ were tested. Model evaluation consisted of plotting model predictions *vs* observed CD19+ lymphocyte counts for the population and for individual patients, relative standard error (RSE) of model parameters, and visual predictive check (VPC). For each VPC, 1000 simulations from the model were conducted.^13^ It was visually assessed whether the median, 2.5th and 97.5th percentiles of observed data lay within the 95% confidence interval of individual percentiles of simulated data.

### 2.3 Dose regimen simulation

Based on the K‐PD model, the time course of CD19+ lymphocyte suppression from baseline was simulated from the following dose regimens: 2 infusions of 750 mg/m^2^ given 2 weeks apart, 4 weekly infusions of 375 mg/m^2^, a single infusion of 750 mg/m^2^, and a single infusion of 375 mg/m^2^.

### 2.4 Nomenclature of targets and ligands

Key protein targets and ligands in this article are hyperlinked to corresponding entries in http://www.guidetopharmacology.org, the common portal for data from the IUPHAR/BPS Guide to PHARMACOLOGY.^14^

## 3 RESULTS

### 3.1 Patient demographics

We collected 258 measurements of CD19+ lymphocyte counts from 39 children with 8 individual autoimmune diseases, with 22 children diagnosed with systemic lupus erythematosis. Demographics of the population are summarized in Table 1. In total, 108 were reported as zero and so replaced with 5 × 10^6^/L (LLOQ/2).

**Table 1:** Demographics of paediatric patients in the current study

Variable	Value/median [range]
No. of patients	39
No. of observations	258
Age (y)	13.2 [3.9–18]
Sex (m/f)	5/34
Rituximab dose (mg)	1000 [413–1000]
CD19+ lymphocytes (10^6^/L)	10 [0–1560]
Baseline CD19+ lymphocytes (10^6^/L)	170 [10–940]
% received methotrexate	25.6
% received cyclophosphamide	43.6
% received azathioprine	33.3
% received mycophenolate	48.7
% received prednisolone	100
% received methylprednisolone	100
% received tacrolimus	5.1
Diagnosis	
ASPM	3
JDERM	4
JIA	1
LOS	1
MPA	1
PAN	1
SLE	22
GPA	6

ASPM, anti‐SRP positive myositis; JDERM, juvenile dermatomyositis; JIA, juvenile idiopathic arthritis; LOS, lupus overlap syndrome; MPA, anti‐neutrophil cytoplasmic antibody‐positive vasculitis; PAN, polyarteritis nodosa; SLE, systemic lupus erythematosus; GPA, granulomatosis with polyangiitis.

### 3.2 Population K‐PD modelling

The M3 method was unstable and successful minimization was not achieved. The final structural model therefore employed the M6 method with LLOQ/2 to handle the zero counts, with a proportional residual error model best describing residual variability. Table 2 summarises the parameter estimates of the K‐PD model. The elimination rate constants of rituximab (k_e_) and CD19+ B cells (k_out_) suggested half‐lives of 19 days and 35 days, respectively. Methotrexate was estimated to increase the maximum cell killing effect of rituximab by 66%, while the additional effect was estimated to be 38% for cyclophosphamide. The comedication effect of azathioprine or mycophenolate mofetil, and the effect of age and sex were all not found to be significant.

**Table 2:** Parameter estimates from the kinetic–pharmacodynamic model

Parameter	Estimate (RSE%)	BSV% (RSE%)
k_e_ (/day)	0.036 (22.7)	73.88 (51.7)
Baseline (10^6^/L)	266.4 (37.8)	98.13 (26.9)
k_out_ (/day)	0.02 (41.0)	89.61 (108.3)
E_max_	35.2 (11.8)	‐
ED_50_ (mg)	0.81 (190.3)	‐
cov_MTX_	0.66 (37.4)	‐
cov_CYC_	0.38 (74.4)	‐
Prop residual error (cv%)	59.3 (14.4)	‐

k_e_, elimination rate constant of rituximab; baseline, CD19+ lymphocyte counts at baseline; k_out_, death rate constant of CD19+ lymphocytes; E_max_, maximum cell killing effect of rituximab; ED_50_, rituximab dose at 50% of maximum effect; cov_MTX_, proportional increase in E_max_ with methotrexate; cov_CYC_ proportional increase in E_max_ with cyclophosphamide; Prop residual error (cv%), coefficient of variation for proportional variability; RSE, relative standard error; BSV, between‐subject variability.

**Figure 2:**
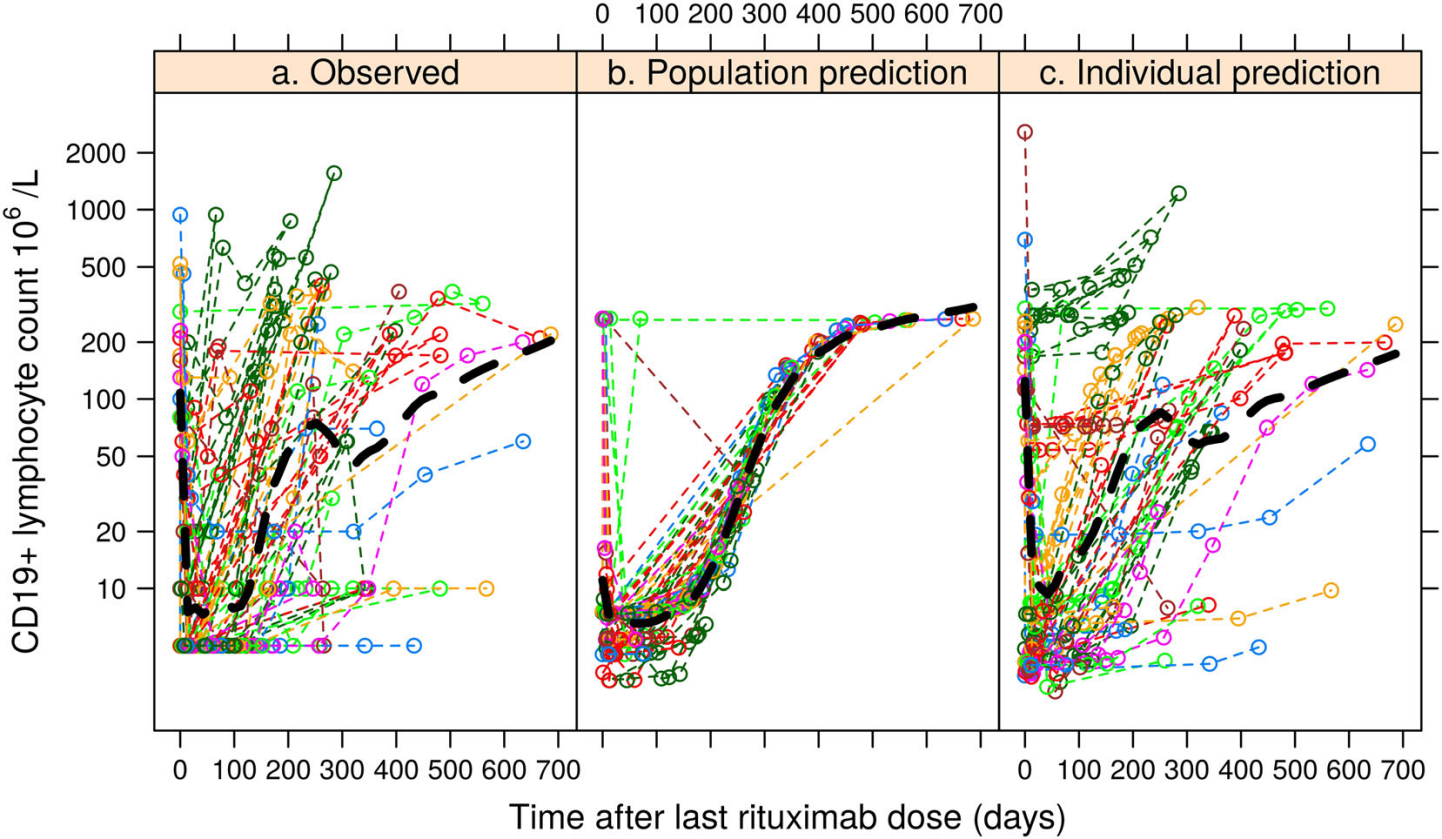
Comparison of (a) observed CD19+ counts with time after the last rituximab dose with (b) population model predictions for a typical individual, and (c) individual model predictions based on empirical Bayesian estimates of the individual model parameters (black dashed line: locally estimated scatterplot smoothing)

**Figure 3:**
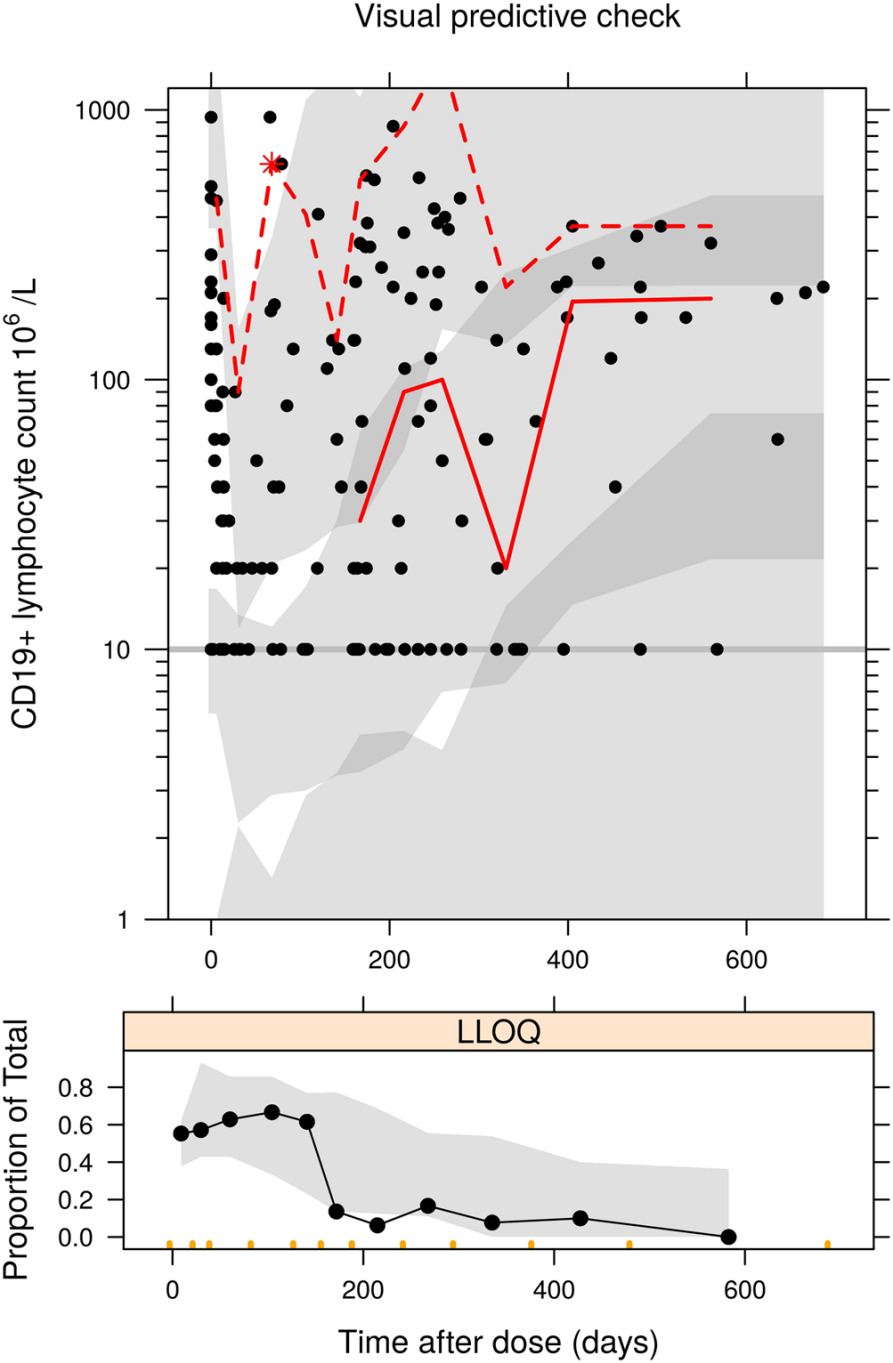
Upper panel: visual predictive check of the kinetic–pharmacodynamic model vs time after dose; lower panel: proportion of observations at each time point below the limit of detection (black circle and line: observed data, grey band: 95% prediction interval of 2.5^th^, 50^th^ and 97.5^th^ percentiles of predicted data, red line: observed 50^th^ and 97.5^th^ percentiles). LLOQ, lower limit of quantification

A comparison of the observed data plotted against time after last rituximab dose compared with the population and individual predictions is given in Figure 2 and a VPC including prediction of the fraction of data below 10 × 10^6^/L is given in Figure 3. Further diagnostic plots, and NONMEM code of the final model are provided in the supplementary material.

### 3.3 Dose regimen simulation

**Figure 4:**
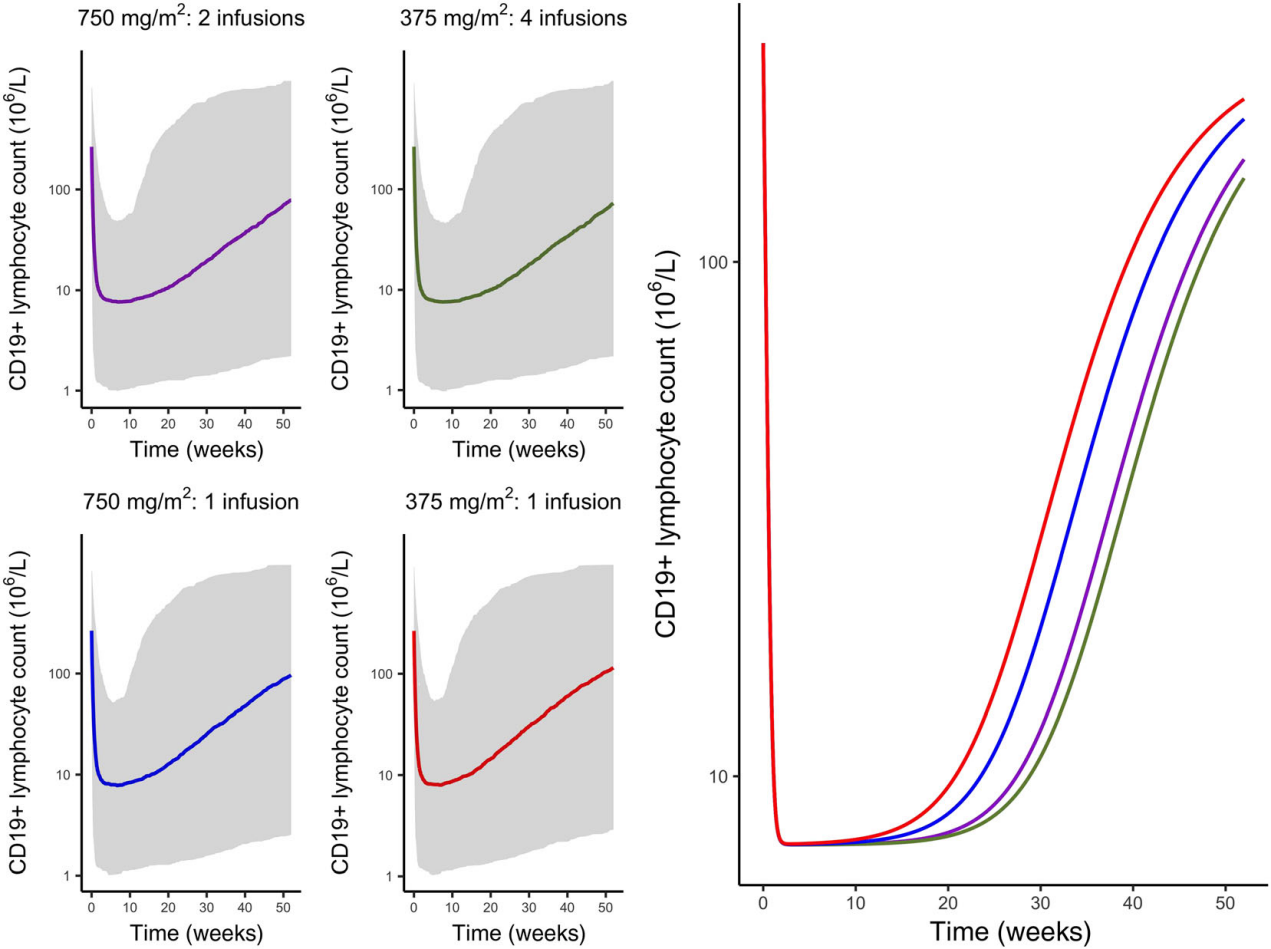
Simulated profiles of suppression of CD19+ lymphocytes after variable dosing regimens of rituximab (purple: 2 infusions of 750 mg/m^2^ given 2 weeks apart, green: four weekly infusions of 375 mg/m^2^, blue: a single infusion of 750 mg/m^2^, red: a single infusion of 375 mg/m^2^, grey band: 95% prediction interval)

Simulations of the time course of CD19+ lymphocytes for the 4 dose regimens are shown in Figure 4, where a single infusion of 375 mg/m^2^ produced mostly identical effect on CD19+ lymphocyte suppression within 10 weeks and the effect remained similar after 6 months, in comparison to the highest suppression achieved by 4 weekly 375 mg/m^2^ infusions (6.2% *vs* 3.3% of baseline CD19+ lymphocyte counts after 6 months). The return of CD19+ lymphocytes, however, occurred more rapidly with lower rituximab doses. Simulations of suppression time to reach lymphocyte counts of 10 × 10^6^/L or 100 × 10^6^/L for patients with a normal baseline CD19+ count of greater than 200 × 10^6^/L are given in Table 3.

**Table 3:** Simulated suppression time to recovery to 10 × 10^6^/L or 100 × 10^6^/L (95% prediction interval) in weeks for patients starting with normal CD19+ counts receiving rituximab at 4 doses

Dosing	Duration (95%PI) below 10 × 10^6^/L (weeks)	Duration (95%PI) below 100 × 10^6^/L (weeks)
750 mg/m^2^: 2 infusions	26.0 (6.4–94.0)	42.7 (11.3–182.5)
375 mg/m^2^: 4 infusions	27.2 (7.6–95.2)	44.1 (12.9–183.7)
750 mg/m^2^: 1 infusion	21.4 (4.3–82.5)	38.6 (9.1–167.8)
375 mg/m^2^: 1 infusion	18.0 (3.7–71.6)	35.7 (8.2–154.2)

PI, prediction interval.

## 4 DISCUSSION

To our knowledge, this is the first study to investigate and quantify the PD of rituximab on CD19+ lymphocytes in paediatric patients. The proposed K‐PD model with turnover mechanism gave a good description of the life span of CD19+ lymphocytes after rituximab treatment (Figures 2, 3), with CD19+ lymphocyte counts measured from children with multiple autoimmune diseases. Our major finding is that a similar peripheral blood CD19+ lymphocyte response could be achieved using lower doses than at present, with a single 750 mg/m^2^ dose producing >80% of the CD19+ lymphocyte suppression time as doubling the dose (Table 3). Since dosing also depends on other clinical measures (including adverse events such as opportunistic infection; off target effects such as neutropenia; and impact on disease activity) that were not available in our data, whether dose reductions are feasible based on these requires further investigation. Another use of our model could be in assessment of the effect of rituximab biosimilars in paediatric populations.

Both methotrexate and cyclophosphamide were found to increase the maximum killing effect significantly, and age and sex did not affect the individual difference in CD19+ lymphocyte suppression following rituximab treatment. Through simulations, a single infusion of lower rituximab dose was able to suppress CD19+ lymphocytes to a mostly identical extent within 10 weeks and the suppression effect remained similar within 6 months (Figure 4).

We did not measure rituximab concentrations in the study, and hence modelled a dose response, the so‐called K‐PD approach.^15^ However, our model did estimate an apparent decay in rituximab effect, which it could be hypothesized might be similar to rituximab's half‐life. In adult patients with rheumatoid arthritis, a 2‐compartment PK model with first‐order elimination described the time course of rituximab serum concentrations,^16^ with the elimination half‐life reported to be 19.7 days. In patients with non‐Hodgkin lymphoma, a 2‐compartment model with time‐varying clearance was developed to account for the decrease in capacity of the IgG‐mediated clearance pathway and the elimination half‐life of rituximab was 6.1–52 (median 22) days.^17^ A further study in chronic lymphocytic leukaemia estimated a median half‐life of 28.3 days (range: 14–49 days).^18^ In our study, the estimated elimination half‐life of rituximab was 11.1–73.7 (median 19.3) days and the value was consistent with these literature findings (Table 2). Collecting PK data of serum concentrations may be challenging in some settings, for example due to the fact that blood samples may be required after the patient is discharged, or complex assay methodology required for biological drug plasma quantification may not be available. We have shown that, for rituximab, the K‐PD modelling approach is able to uncover underlying PK information in the absence of PK data.

The turnover of CD19+ lymphocytes was characterized by zero‐order cell proliferation and first‐order decay over time as a simplified representation of the underlying sequential development steps of B cell maturation in bone marrow, mitigation into spleen as transitional B cells, and differentiation into mature B cells.^19^ The elimination half‐life of B cells was estimated to be 34.7 days (Table 2), which was consistent with findings from the literature, although large variability was seen for individual patients ranging from 18.3 to 333 days. Fulcher and Basten^20^ reviewed experimental studies in mice and reported first‐order exponential decay of B cells over time with a half‐life between 5 and 6 weeks for most peripheral B cells. Macallan et al. investigated in vivo B cells in healthy adults and reported variable B cell elimination half‐lives ranging between 1 and 9 weeks.^21^ Based on the turnover mechanism of CD19+ lymphocytes, asymptotic increase on cell production rate over age was examined, although the changeover age was not found to be statistically significant. From infancy to adulthood, cell growth may differ between females and males due to oestrogen^10^ but the sex difference was not a significant covariate in our study.

Comedication effects of 4 immunosuppressants, including methotrexate, cyclophosphamide, azathioprine and mycophenolate, were considered in the current study and only methotrexate and cyclophosphamide were found statistically significant; both small‐molecule comedications cause immunosuppression via the inhibition of cell replication and transcription that ultimately lead to apoptosis of malignant cells.^22^ This mechanism of action is different from rituximab that depletes B cells via cell‐mediated cytotoxicity and therefore methotrexate and cyclophosphamide were considered to enhance the elimination of CD19+ lymphocytes individually and independently. In the K‐PD model, the independent roles of methotrexate and cyclophosphamide on killing B cells were tested on both E_max_ and ED_50_ parameters (apparent maximum effect and apparent potency). It was found that methotrexate and cyclophosphamide increased apparent maximum effect by 66 and 38%, respectively (Table 2); however, the comedication effect from either small molecule was overall negligible regarding the suppression of CD19+ lymphocytes over time.

A limitation of our work was the relatively limited sample size. A large proportion of our data were below the limit of detection, but the rather simple solution of replacing these values with half the limit worked well in that model simulated fractions matched those observed (Figure 3). Whilst we estimated parameters consistent with literature values, suggesting that the data were reliable and our model valid, the study was perhaps under‐powered to detect some covariate effects. For example, children may undergo maturation and development over time and the age‐dependent cell growth, i.e. total CD19+ B cell counts within the lymphocytes decrease with age,^23^ which may be observed and quantified in a larger cohort covering a wider range of age groups. Due to the small sample size large between‐subject variability values, but remaining within a reasonable range, were reported and concomitant drugs were found to account for a proportion of the variability in cell death rate (k_out_) (Table 2). Given the limited sample size low relative standard error values were reported for model parameters suggesting precise parameter estimates, except for ED_50_ due to lack of observed information on residual doses within the body over time (Table 2). If we assume a plasma volume of 1.5 L for a typical child in our study, an ED_50_ of 0.81 mg corresponds to a plasma concentration of approximately 0.54 μg/mL, whereas *in vitro* studies show EC_50_ values in the range 2–10 μg/mL.^24^ Therefore, despite the fact that this parameter is estimated with large uncertainty, its value is not much outside the plausibly expected range. Another limitation of our work was the lack of measurements of CD19+ lymphocytes in tissues. We have therefore assumed that depletion of CD19+ lymphocytes are representative of tissue levels, which may or may not be the case. This is a further reason that future work should investigate clinical outcomes in addition.

Simulations of the time course of CD19+ lymphocytes following various dose regimens of rituximab were conducted to determine whether other dose schemes may be used. Upon finding a low ED_50_ value, we hypothesized that a similar long‐term effect could be achieved using a lower dose. We found that a single infusion of 750 mg/m^2^ or perhaps even 375 mg/m^2^ could be used for treating children with autoimmune diseases while retaining similar treatment effect as observed by CD19+ lymphocyte reduction within 6 months (Figure 4); meanwhile the lowering rituximab dose would yield reduced treatment costs and possibly reduced infection risk. The recovery of CD19+ lymphocytes, however, is likely to occur more rapidly with lower rituximab doses and the benefit–cost balance of rituximab needs to be considered in clinical practice.

Ultimately, the findings from our study may be used as underlying prior PKPD knowledge against which biosimilars of rituximab could be compared using PK if serum measurements are available for biosimilars or using the PD endpoint as in the current study. Such a PD bioequivalence could inform rituximab biosimilar dosing in the paediatric population, and therefore allow more economic access to therapy in children with autoimmune diseases.

## 5 CONCLUSIONS

The current study quantified the effect of rituximab on reducing CD19+ lymphocytes in paediatric patients with autoimmune diseases through population K‐PD modelling. Based on simulations from the K‐PD model, a lower dose than the current standard regimen should provide a similar effect on CD19+ lymphocyte suppression at reduced expense in children, although the impact of lower doses of rituximab on other biomarker or clinical measurements is unknown. Ultimately, the findings may also contribute to the investigation of bioequivalence and dose establishment of rituximab biosimilars, and the design of future therapeutic trials of B cell‐depleting agents in paediatric patients with autoimmunity.

## COMPETING INTERESTS

The authors declare no conflict of interest.

## CONTRIBUTORS

D.E. and J.F.S. conceived the study. I.C., H.Y. and A.S. compiled the data and contributed to model development by S.P. and J.F.S. P.B., S.M. and D.E. were members of the clinical team responsible for prescribing rituximab and I.C. was a member of the clinical pharmacy team. All authors contributed to manuscript writing, which was led by S.P.

## Supporting information

Figure S1: Normalised prediction distribution errors (NPDE) vs time
