# Supplementary material for: Pharmacodynamics of rituximab on B lymphocytes in paediatric patients with autoimmune diseases
Source: Br J Clin Pharmacol. 2019 Jun 20;85(8):1790–7. doi: 10.1111/bcp.13970 (PMC6624401; doi:10.1111/bcp.13970)
Supplement: Supplementary file 1 — Figure S1: Normalised prediction distribution errors (NPDE) vs time [file BCP-85-1790-s001.pdf]

SUPPLEMENTARY MATERIAL: Pharmacodynamics of rituximab on B lymphocytes in paediatric patients with autoimmune diseases

Contents

1 Further goodness of fit plot **1**

2 NONMEM model file with values set to final estimates **2**

1 Further goodness of fit plot

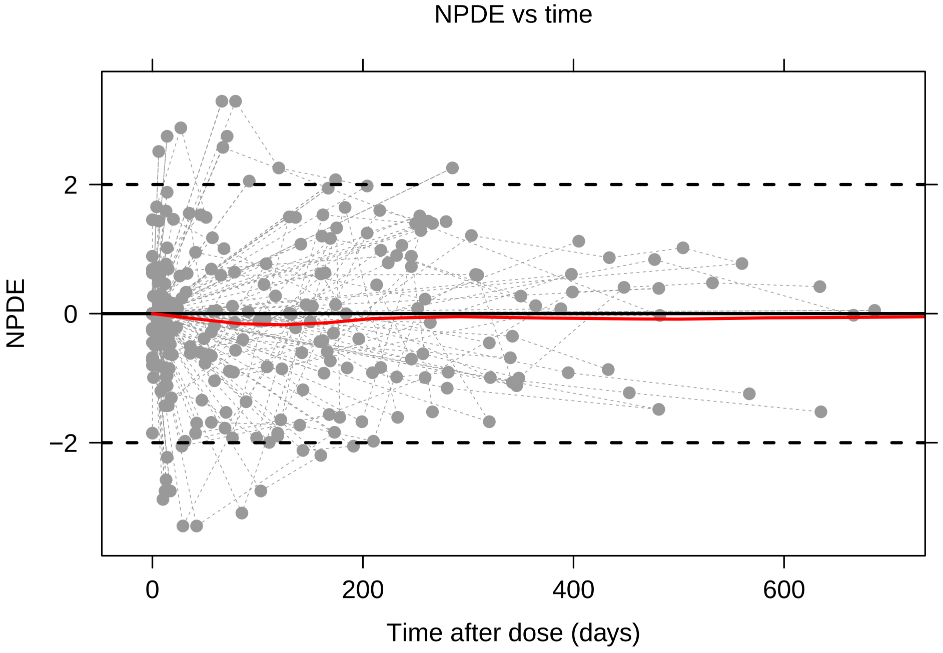

Figure S1: Normalised Prediction Distribution Errors (NPDE) versus time

2 NONMEM model file with values set to final estimates

```
;; 1. Based on:
;; 2. Description: Rituximab KPD modelling for the time course of B cell count
;; AUTHOR: SP
;; DATE: 14-MAR-2018
;; UNITS: AMT:MG, TIME:DAY, DV:10^6/L
;; MODEL STRUCTURE: KPD (KE ON DOSE)
;; ERROR MODEL: PROP
;; COVARIATE: MTX AND CTX ON EMAX
$PROBLEM Rituximab KPD modelling for the time course of B cell count

$INPUT ID TIME CMT AMT DV EVID MDVO SEX AGE AGEY AZA CSA CTX CTX_OLD DXM
       FCT HCT MTX MEP MMF PRE TAC DGNS TAD BLQ

$DATA rituximab_181205.csv IGNORE=@

$SUBROUTINE ADVAN6 TOL=6
$MODEL      COMP=(DOSE) COMP=(BCELL)
$PK
;------Model parameters------
KE   = THETA(1) * EXP(ETA(1))
BSL  = THETA(2) * EXP(ETA(2))
KOUT = THETA(3) * EXP(ETA(3))
;------Covariates------
CCOV = 0
IF (CTX.EQ.1) CCOV = THETA(7)
CCOV1 = 0
IF (MTX.EQ.1) CCOV1 = THETA(8)
;------Drug effect------
EMAX = THETA(4) * (1 + CCOV) * (1 + CCOV1)
ED50 = THETA(5)
;------Rate constants and initialise------
KIN = BSL * KOUT
A_0(2) = BSL
;------Proportional error------
CV = THETA(6)

$DES
DADT(1) = -KE * A(1)
DG      = (EMAX * A(1)) / (ED50 + A(1))
DADT(2) = KIN - KOUT*(1 + DG) * A(2)

$ERROR
IPRED = A(2)
SD    = SQRT(0.0001 + CV*IPRED**2)
Y     = IPRED + SD*EPS(1)
IRES  = DV-IPRED
IWRES = IRES / SD

$THETA
 (0,0.0359064) ; KE
 (0,266.449)   ; BSL
```

```
 (0,0.0160064) ; KOUT (per day)
 (0,35.1538)   ; EMAX
 (0,0.811751)  ; ED50 (mg)
 (0,0.351255)  ; CV
 (-1,0.381171) ; CCOV
 (-1,0.657277) ; CCOV1
$OMEGA
 0.54598   ; BSV_KE
 0.96308   ; BSV_BSL
 0.803053  ; BSV_KOUT
$SIGMA  1  FIX
$ESTIMATION METHOD=1 INTER MAXEVALS=9999 POSTHOC NOABORT PRINT=1
$COVARIANCE UNCONDITIONAL PRINT=E MATRIX=S
$TABLE      ID TIME AMT EVID IPRED CWRES IWRES TAD NPDE ESAMPLE=1000
            ONEHEADER NOPRINT FILE=sdtab20
$TABLE      ID KE BSL KOUT EMAX ED50 ETA1 ETA2 ETA3 NOPRINT NOAPPEND
            ONEHEADER FILE=patab20
$TABLE      ID AGE AGEY NOPRINT NOAPPEND ONEHEADER FILE=cotab20
$TABLE      ID GENDE AZA CSA CTX DXM FCT HCT MTX MEP MMF PRE TAC BLQ
            NOPRINT NOAPPEND ONEHEADER FILE=catab20
```
